# Establishment of OPG Transgenic Mice and the Effect of OPG on Bone Microarchitecture

**DOI:** 10.1155/2013/125932

**Published:** 2013-03-27

**Authors:** Ying Wu, Jianghua Liu, Hui Guo, Qiong Luo, Ziying Yu, Eryuan Liao, Xuyu Zu

**Affiliations:** ^1^Department of Metabolism and Endocrinology, The First Affiliated Hospital of University of South China, Hengyang, Hunan 421000, China; ^2^Institute of Clinical Medicine, The First Affiliated Hospital of University of South China, Hengyang, Hunan 421000, China; ^3^Institute of Metabolism and Endocrinology, The Second Xiangya Hospital, Central South University, Changsha, Hunan 410011, China

## Abstract

Osteoprotegerin (OPG) plays a determinant role in regulating bone metabolism, but the effect of OPG on bone microarchitecture needs to be further elucidated. We attempted to construct pCI-hOPGp-mOPG vector containing human OPG promoter and FLAG tag and to microinject vector into fertilized zygotes from C57BL/6J × CBA mice to prepare transgenic mice. The OPG transgenic positive mice were identified by PCR and western blotting. Twelve-week-old OPG transgenic mice (OPG-Tg mice) and wild-type mice (WT mice) were utilized in the study of bone microarchitecture. Microcomputed tomography (micro-CT) data showed that compared with WT mice, the tibia of OPG-Tg mice showed an increased volumetric BMD (vBMD), tissue BMD (tBMD), trabecular thickness (Tb.Th), and trabecular number (Tb.N), and a decreased trabecular separation (Th.Sp) (*P* < 0.05) . The cortical bone microarchitecture parameters, such as cortical area (Ct.Ar), cortical thickness (Ct.Th), cortical BMD (Ct.BMD), cortical BMC (Ct.BMC), BMD, and BMC of femur, were increased, and the inner perimeter (In.Pm) was decreased, in OPG-Tg mice, compared to those in WT mice (*P* < 0.05). The established OPG transgenic mouse model could be valuable for further studying the biological significance and gene regulation of OPG in vivo.

## 1. Introduction

The skeleton is in a dynamic state, being continually degraded and renewed in a tightly regulated remodeling process that involves a complex network of systemic hormones and local factors. Among the local signaling factors implicated in this process is OPG/RANK/RANKL system. OPG, a secreted member of the tumor necrosis factor receptor superfamily, has been identified as an osteoblast-derived regulator of bone resorption and bone mass, and it is implicated in the pathogens of postmenopausal osteoporosis and other metabolic bone diseases. OPG acts by neutralizing RANKL, an essential cytokine required for osteoclast formation and activation [1]. Prior studies have showed that OPG is not only an important regulatory factor of bone growth, bone modeling, and bone remodeling [2, 3], but also an intermediary factor of a wide variety of hormones, cytokine, and growth factors which are involved in regulating bone metabolism [4]. In vitro, osteoclast differentiation which is induced by 1,25-(OH)_2_D_3_, PTH, PGE_2_, and IL-4 is blocked by OPG in a dose-dependent manner [5, 6]; in vivo, recombinant OPG increases significantly bone mass and bone mineral density of lumbar spine and femoral of ovariectomized rat [7]. In clinical trials, recombinant OPG can be used for postmenopausal women for the treatment of osteoporosis [8]. These data suggested that OPG could act as a key factor in the regulation of bone mass and implied a utility for OPG in the treatment of metabolic bone disease. In addition to the effect on bone tissue, OPG was also reported to influence cardiovascular [9] and immune system [10]. Therefore, a suitable animal model with OPG overexpression is needed for the further insight into the implication of OPG in metabolic bone disease.

Transgenesis is a process where foreign genes are inserted into an animal's DNA, and it is popular in the biomedical science. Scientists create, so-called transgenic animals to investigate disease treatments, produce natural material, and expand scientific knowledge. Transgenic mice are the most widely used as experimental models to unravel gene phenotype and they are valuable tools for biomedical research [11]. In our previous study, OPG knockout mice showed increased bone remodeling, deceased bone density, and reduction in bone strength [12]. In the present work, we firstly created OPG transgenic mice containing human OPG promoter and FLAG tag. Furthermore, we obtained the quantitative data of bone microarchitecture in OPG transgenic mice for the first time.

## 2. Materials and Methods

### 2.1. Animals

C57BL/6J mice (WT mice) were purchased from the Shanghai Research Center for Biomodel Organisms. All studies were performed with the approval of the Experimental Animal Committee at the University of South China. All the animals were housed under specific pathogen-free conditions (22°C, 12 h/12 h light/dark, 50%–55% humidity) with free access to food pellets and tap water in the experimental animal center of the University of South China.

### 2.2. Construction of Gene Targeting Plasmid

The liver RNA extracted from C57BL/6J mice was used to perform two PCR amplification using Pfu PCR enzyme to obtain mOPG cDNA sequence. Then mOPG cDNA sequence and pCI-NEO-OPG-LacZ plasmid which contained human OPG promoter were double digested by using Not I and Sal I. The digested fragments were connected by using T4 ligase, and then the connected fragments were transferred into competent cells from Escherichia coli and tiled in LB plate. Monoclonal bacteria were selected and the pCI-hOPGp-mOPG plasmid was confirmed by enzyme digestion and sequence reaction. The vector was digested by using BglII/KpnI to obtain linear DNA fragment for further transgenic mice establishment.

### 2.3. Generation of Transgenic Mice

Six–eight-week C57BL/6J × CBA female mice were selected to perform ovarian stimulation and subsequently mated with healthy male mice overnight. The female mice which have vaginal pessary were selected as receptor for embryo transplantation, and then fertilized eggs were obtained from the receptors by surgery. Linear DNA was injected into fertilized eggs (pronuclear stage) by using an inverted microscope, micromanipulation equipment, and injection/holding devices. To generate OPG-Tg mice, 15~30 recombinant cells were injected into C57BL/6J blastocysts and then transferred into uterus of pseudopregnant mice. After young mice were born, progeny was screened for the presence of the OPG by PCR and western Blot.

### 2.4. Genotype Identification

Genomic DNA was extracted and purified from the tail tissue of each mouse. To genotype the OPG-Tg mice, PCR was performed using the following primers: OPG-JD-1 : 5-TCA AAG GCA GGC GAT ACT-3 and OPG-JD-2 : 5-CAA TGT CTT CCT CCT CAC TGT-3.

### 2.5. OPG Protein Expression Analysis

The mice were sacrificed after 12 weeks, and proteins were extracted from the livers. The OPG protein levels in the mice were detected by performing a western blot assay using rabbit anti-FlAG polyclonal antibodies [13, 14].

### 2.6. Micro-CT

The right tibia was fixed with 4% paraformaldehyde for 24 h and subsequently washed with 10% saccharose solution for 12 h. Micro-CT scanning was performed using the GE explore Locus SP system. The proximal tibia was selected as the region for scanning by the fluoro method. The center of rotation and the CT value were artificially modified and the entire results obtained for the samples scanned with an isotropic resolution of 8.0 × 8.0 × 8.0 *μ*m voxels were reconstructed after completion of the scanning. For the cancellous bone analysis, bone tissue of 0.8 mm thickness at a distance of 0.16 mm from the distal end of the growth plate was selected from the image as the region of interest (ROI) in order to perform a three-dimensional reconstruction of 8.0 × 8.0 × 8.0 *μ*m voxels; image information was obtained based on the automatic domain value provided by the computer in order to accomplish binary conversion of the image. Selected areas of cancellous bone within the ROI were demonstrated three dimensionally. For the cortical bone analysis, bone tissue, 1.0 mm in length and 0.16 mm in thickness, from the middle diaphysis of the tibia was selected as the ROI from the reconstructed image to perform three-dimensional reconstruction of 8.0 × 8.0 × 8.0 *μ*m voxels, and image information was obtained based on the automatic domain value yielded by the computer. The 2.0^+^ABA Microview software of the micro-CT system was applied to perform quantitative analysis on the reconstructed images.

### 2.7. DXA Scanning and Image Analysis

The left femur was fixed onto the scanning table along the longitudinal axis and subsequently scanned by dual-energy X-ray absorptiometry (DXA) using a PIXImus densitometer (GE Lunar, Madison, WI) to determine the BMD and BMC. Scanning parameters are as follows: the scan spacing was 1.0 × 1.0 mm, scanning speed was 60.0 mm/s, and the accuracy was 1.0%.

### 2.8. Statistics

The SPSS 15.0 statistical software was used for statistical analysis. Data are reported as mean ± SD. Data were analyzed by analysis of variance followed by the Student-Newman-Keuls Multiple Comparisons test, and statistical significance was considered when *P* was less than 0.05.

## 3. Results

### 3.1. Construction of Gene Targeting Plasmid

**Figure 1:**
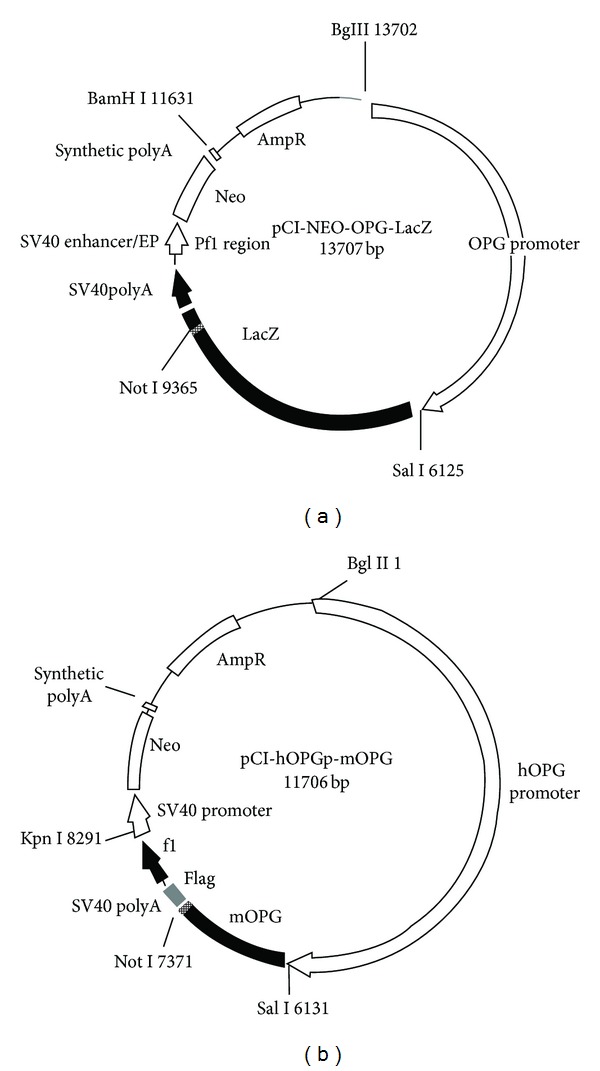
Structure scheme of plasmids. (a) PCI-NEO-OPG-LacZ plasmid profile. (b) PCI-hOPGp-mOPG plasmid profile.

**Figure 2:**
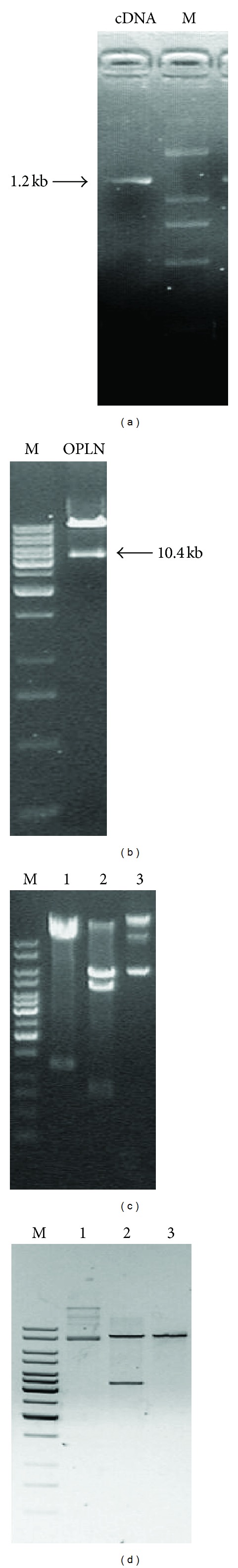
Recombinant PCI-hOPGp-mOPG plasmid construction and plasmid linearization. (a) mCPG DNA was digested by Not I and Sal I. (b) PCI-NEO-OPG-LacZ was digested by restriction enzymes Not I and Sal. (c) Identification of the digested pCI-hOPG-mOPG.M:Marker1Kb Ladder. 1: pCI-hOPG-mOPG was digested by NotI and SalI; 2: pCI-hOPG-mOPG was digested by EcoRI; 3: pCI-hOPG-mOPG plasmid. (d) linear pCI-hOPGp-mOPG plasmid DNA.M:1KbLadder; 1: pCI-hOPG-mOPG palsmid; 2: pCI-hOPG-mOPG digested by BglII/KpnI; 3: pCI-hOPG-mOPG digested by BglII/KpnI was recovered.

The liver RNA extracted from C57BL/6J mice was utilized to perform RT-PCR to obtain OPG cDNA sequence. OPG cDNA sequence and pCI-NEO-OPG-LacZ plasmid (Figure 1(a)) containing human OPG promoter were double digested by using Not I and Sal I, and a 1.2 kb fragment from mOPG cDNA and a 10.4 kb fragment from pCI-NEO-OPG-LacZ were observed (Figures 2(a) and 2(b)). The recombinant pCI-hOPGp-mOPG plasmid (Figure 1(b)) showed a 1.2 kb fragment and a 10.4 kb fragment with the digestion of Not I and Sal I. 766 bp, and 5354 bp and 5586 bp fragments were observed after the digestion EcoR (Figure 2(c)). The recombinant pCI-hOPGp-mOPG plasmid was linealized by the digestion with BglII and KpnI to obtain two DNA fragments of 8291 bp and 3411 bp, and the 8291 bp linearized pCI-hOPGp-mOPG fragment was microinjected into mouse fertilized egg cells (Figure 2(d)).

### 3.2. Positive Transgenic Mice Identification

**Figure 3:**
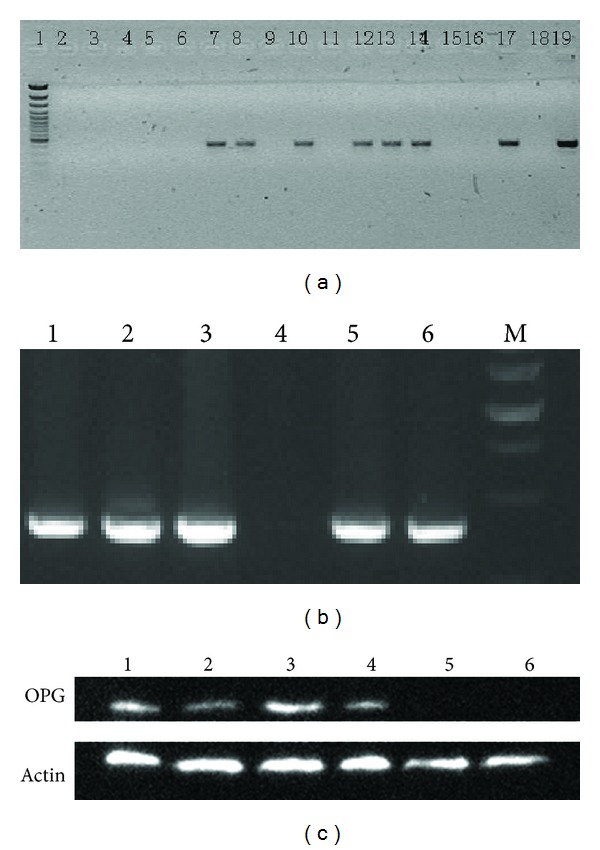
Identification of genotypes of OPG-Tg mice. (a) Identification of OPG genotypes in OPG-Tg founder mice; 1: GenenRuler 100 bp DNA Ladder; 2–17: mice samples; 18: ddH2O; 19: positive control. (b) Identification of young mice genotypes. M: GenenRuler100 bp DNA Ladder; 1–3: young pup samples; 4: ddH_2_O; 5-6: positive control. (c) OPG-Tg mice liver's western blot examination. 1–4: OPG-Tg mouse; 5: WT mouse; 6: H_2_O.

During the course of transgenic mice preparation, total 426 injection eggs were transplanted into 17 pseudopregnant female mice tuba, and 12 female mice of them were shown to be pregnant. Total 69 mice were born and 7 positive transgenic mice were confirmed by PCR screening. Furthermore, genomic DNA extracted from positive mice and WT mice tails was used for double-blinded PCR to further confirm the seven positive transgenic mice (Figure 3(a)). The transgenic founder female mice were fed together with WT male mice in the cage rearing at the ratio of 1 : 1, and transgenic founder male mice were fed with WT female mice in cage at the ratio of 1 : 3. To identify the genotype of the offspring, special primers across the hOPG promoter and mOPG were used for the PCR screening, and as shown in Figure 3(b), no band was detected in WT mice and one 385 bp band was observed in OPG-Tg mice. To further confirm the genotype of the transgenic mice, the proteins were extracted from the mice livers and western blot was performed using rabbit anti-FLAG polyclonal antibodies. As shown in Figure 3(c), the FLAG tag could not be detected in the WT mice, whereas a 60-kD OPG protein containing FLAG tag could be detected in the OPG-Tg mice.

### 3.3. BMD and BMC Measurement

The whole left femur was scanned by DXA to determine the BMD and BMC. It was found that OPG-Tg mice showed increases of 42.3% and 151.7% of overall femoral BMD and BMC, respectively, compared to those of WT mice (Table 1).

### 3.4. Micro-CT

**Figure 4:**
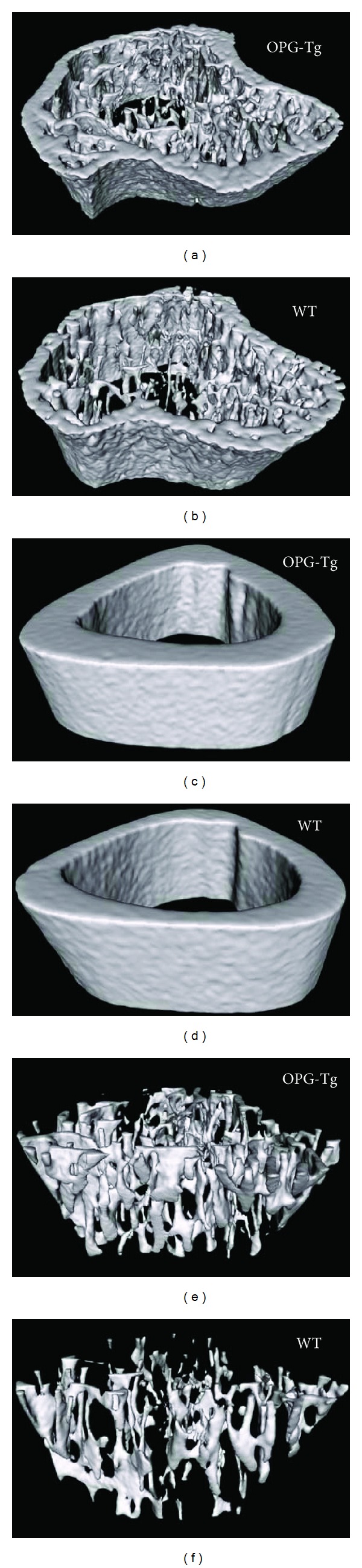
Three-dimensional micro-CT images of the right tibia from two types of mice. (a) Image of the right tibia of OPG-Tg mice; (b) image of the right tibia of WT mice; (c) image of the cortex of OPG-Tg mice; (d) image of the cortex of WT mice; (e) image of the trabecular of OPG-Tg mice; (f) image of the trabecular of WT mice.

To observe the effect of OPG on bone microarchitecture, micro-CT was used. As shown in Figure 4, compared with WT mice, cancellous bone trabecular number of OPG-Tg mice increased significantly; distribution of bone trabecula in OPG-Tg mice became denser and more continuous and trabecular separation of OPG-Tg mice became smaller. Micro-CT scanning also showed that the OPG-Tg mice had an increase of 40.6%, 11.7%, 60%, and 62.1% for vBMD, tBMD, Tb.Th, and Tb.N, respectively, and a decrease of 47.4% for Th.Sp compared to those of WT mice (Table 2). The cortical bone structural parameter also showed difference between OPG-Tg mice and WT mice. OPG-Tg mice showed obviously thicker cortical bone and narrower lumen (Figure 4). It was also confirmed that the OPG-Tg mice mice showed an increase of 81.0%, 132.9%, 15.7%, and 118.2% for Ct.Ar, Ct.Th, Ct.BMD, and Ct.BMC respectively, and a decrease of 15.4% for In.Pm compared to those of WT mice. No significant outer perimeter (Ot.Pm) difference was observed between OPG-Tg mice and WT mice (Table 3).

## 4. Discussion

OPG, a novel secreted member of the TNFR superfamily, acts as a soluble receptor antagonist of RANKL by preventing it from binding to RANK, and the interaction of RANKL and RANK has been shown to be required for osteoclast differentiation. Any dysregulation of their respective expression leads to bone tumor-associated osteolysis, immune disease, and cardiovascular pathology. Many researches have proved that OPG can inhibit osteoclast maturation and protect bone from both normal osteoclast remodeling and ovariectomy-associated bone loss, indicating that OPG might be a key determinant in regulating bone metabolism [15]. Firstly, RANKL is a membrane-bound protein of the tumor necrosis factor ligand family that is expressed on the osteoblast cell surface and has been shown to play a major role in osteoclast differentiation along with macrophage colony stimulating factor. RANKL binds to its receptor RANK on hematopoietic cells and initiates a cascade of signaling events that leads to osteoclast differentiation. As a decoy receptor for RANKL, OPG can prevent its interaction with the cognate receptor RANK. OPG has been shown to be a potent inhibitor of bone resorption through interfering osteoclast survival, differentiation, and biological activity in vitro. Secondly, OPG can prevent TAF6 activation with RANK/RANKL, thus inhibiting osteoclast activation and maturation, and this could ultimately result in the reduction of functional osteoclast. Thirdly, OPG can also prevent stroma cell from the interaction with osteoclast, and this inhibitory ability can be protected in the presence of caspase-3 inhibitor, indicating that OPG can prevent bone loss through inducing osteoclast apoptosis [16]. Finally, OPG can also affect the function of osteoclast directly by increasing the protease and trypsin inhibitor expression. In summary, the mechanism of OPG in regulating bone metabolism is complicated and deserves more investigations [17]. OPG was also shown to influence cardiovascular and immune system except for the bone tissue. OPG-deficient mice have shown early onset arterial calcification [18, 19], suggesting an important role of OPG in the protection of blood vessels, whereas recent studies showed that OPG might increase adhesion function of endothelial cell and serum OPG level is associated with atherosclerosis severity. In the immunity system, OPG can regulate dendritic cell differentiation and maturation, affecting lymphocyte development and function [20]. Due to the diverse function of OPG, a suitable animal model is needed for the comprehensive understanding of OPG function.

 Recently, the advances in transgenic technologies have made it possible for the developments of different transgenic animal models. Compared with other animal model, mouse shows higher genome homology with human, so it is widely used as transgenic animal model for biomedical research. Hence, the mouse is unique in offering the possibility to understand genotype-phenotype relationships that are relevant for unraveling the biologic role of the genes in human [11]. OPG-deficient mice exhibited bone loss with an increase in both bone resorption and formation, which just like high bone turnover postmenopausal osteoporosis [21]. In this work, we ligated full-length mouse OPG cDNA sequence with pCI-NEO-LacZ plasmid which contains human OPG promoter to obtain pCI-hOPGp-mOPG vector. Because variety of FLAG-tagged protein can retain their biochemical activity, FLAG tag is widely utilized as screening marker in the development of transgenic cells and animals models [22]. So FLAG tag was added to pCI-hOPGp-mOPG vector to improve the screen efficiency of OPG-Tg mice. Because human OPG promoter has a transcriptional activity without tissue specificity, it could be anticipated that the established OPG transgenic mice could have a widely exogenous expression of OPG in different tissues. In order to avoid possible noise from endogenous OPG in the screening process, specific primers that span from the human OPG promoter to mOPG were designed to improve the correct rate in the OPG-Tg mice screening; moreover, FLAG protein expression in the OPG-Tg mice was also detected to further devoid those transgenic mice without OPG expression due to different genomic context. Since different founder mice had different insertion sites, we got at least two transgenic mice lines to observe their phenotype, and the same phenotypes from different transgenic mice lines were considered as phenotypes of *OPG* gene. 

Micro-CT is an evolving technique that has the ability to measure three-dimensional (3D) bone microstructure in arbitrary orientations in a highly automated, objective, non-destructive manner, allowing great number of samples for unbiased comparisons between controls and the disordered or treated. Compared to two-dimensional image (2D) data of DXA, micro-CT can not only directly observe the 3D images of cortical bone and trabecular bone microstructure, but also make a quantitative analysis of the 3D structure such as trabecular volume, trabecular thickness, number, separation, structure model index, degree of anisotropy, and connectivity, in a model-independent manner. Quantitative analysis of 3D bone microstructure characteristics may improve our ability to understand the pathophysiology of osteoporosis, to test the efficacy of pharmaceutical intervention, and to estimate bone biomechanical properties [23]. So it is very meaningful to measure 3D bone microstructure of OPG transgenic mice to observe the direct effect of OPG on bone mass. Previous research has reported the quantitative analysis of 3D bone microstructure in the OPG transgenic rats [24], and in the present study, we for the first time quantified 3D bone microstructure in OPG transgenic mice. Micro-CT data showed that compared with wild-type mice, the OPG-Tg mice showed increased tibial cancellous bone microstructural parameters such as vBMD, tBMD, Tb.Th, and Tb.N than those in wild-type mice. The cortical bone microarchitecture parameters of tibia such as Ct.Ar, Ct.Th, Ct.BMD, and Ct.BMC in OPG-Tg mice were significantly higher than those in WT mice. The 3D structure diagram of tibia showed that cortical bone appeared thick and luminal appeared narrow in OPG-Tg mice. The 2D image data of DXA also showed that BMD and BMC were significantly improved in OPG-Tg mice. 

## 5. Conclusion

In summary, we for the first time developed OPG transgenic mice model containing human OPG promoter and FLAG tag, and a quantitative analysis of the 3D bone microarchitecture in OPG transgenic mice was also carried out. Our results show that the bone mass of cancellous and cortical bone in OPG transgenic mice is significantly increased. The established OPG transgenic mouse model would offer a suitable animal model for further insight into the regulating mechanism of bone metabolism.

## Figures and Tables

**Table 1:** Femur overall bone mineral density and bone mineral content of two types of mice.

	BMD (mg/cm^2^)	BMC (mg)
OPG-Tg	32.3 ± 1.2^a^	7.3 ± 1.1^a^
WT	22.7 ± 0.9	2.9 ± 0.7

Values are means ± SD of 4 mice. ^a^ Compared with WT group, *P* < 0.05.

**Table 2:** Comparison of trabecular bone structural parameter.

	WT	OPG-Tg
vBMD (mg/mm^3^)	207.9 ± 27.6	292.3 ± 30.1^a^
tBMD (mg/mm^3^)	621.7 ± 26.9	694.5 ± 33.1^a^
Tb.Th (mm)	0.015 ± 0.001	0.024 ± 0.002^b^
Tb.N (mm^−1^)	3.11 ± 0.63	5.04 ± 0.82^a^
Tb.SP (mm)	0.175 ± 0.021	0.092 ± 0.010^b^

Values are means ± SD of 6 mice. ^a^ Compared with WT group, *P* < 0.05.

^b^ Compared with WT group, *P* < 0.01.

**Table 3:** Comparison of cortical bone structural parameter.

	WT	OPG-Tg
In.Pm (mm)	2.317 ± 0.098	1.961 ± 0.082^a^
Ot.Pm (mm)	3.677 ± 0.271	3.881 ± 0.541
Ct.Ar (mm^2^)	0.263 ± 0.014	0.476 ± 0.032^b^
Ct.Th (mm)	0.082 ± 0.001	0.191 ± 0.004^b^
Ct.BMD (mg/mm^3^)	1017.3 ± 9.3	1176.8 ± 10.9^b^
Ct.BMC (g)	0.0022 ± 0.0001	0.0048 ± 0.0006^b^

Values are means ± SD of 6 mice. ^a^ Compared with WT group, *P* < 0.05.

^b^ Compared with WT group, *P* < 0.01.
